# Maresin-1 Ameliorates Chronic Unpredictable Stress-Induced Depressive-like Behaviors Associated with Dynamic Modulation of Hippocampal Microglial Activity and TSPO PET Signals

**DOI:** 10.3390/biomedicines14020335

**Published:** 2026-01-31

**Authors:** Anhai Zheng, Tian Qiu, Lei Shi, Lixia Wang, Zhu Xia, Zhiping Peng, Li Kuang, Jiamei Guo

**Affiliations:** 1 Department of Psychiatry, The First Affiliated Hospital of Chongqing Medical University, Chongqing 400016, China; 203807@hospital.cqmu.edu.cn (A.Z.); tianqiu131@sina.com (T.Q.); 800476@hospital.cqmu.edu.cn (L.S.); wlx_ic@163.com (L.W.); 2 Department of Nuclear Medicine, The First Affiliated Hospital of Chongqing Medical University, Chongqing 400016, China; xiazhu87@163.com; 3 College of Basic Medicine, Chongqing Medical University, Chongqing 400016, China; pengzhiping@cqmu.edu.cn; 4 Psychiatric Center, The First Affiliated Hospital of Chongqing Medical University, Chongqing 400016, China

**Keywords:** Maresin-1, depression, CUS, microglia, TSPO, PET/CT

## Abstract

**Background/Objectives**: Maresin-1 (MaR1), a specialized pro-resolving mediator (SPM) derived from omega-3 fatty acids, has demonstrated potent anti-inflammatory and pro-resolving properties. However, its effects on depression-like behaviors and the associated dynamics of neuroinflammation, particularly in the context of chronic stress, are not yet fully understood. This study aimed to investigate the therapeutic potential of MaR1 in a chronic unpredictable stress (CUS) model and to monitor its dynamic effects on neuroimmune activity using longitudinal in vivo imaging. **Methods:** Adolescent male C57BL/6J mice were subjected to a 5-week CUS protocol. Mice exhibiting stable anhedonia were randomized to receive intraperitoneal injections of either MaR1 (5 µg/kg) or vehicle every other day for 4 weeks. During this period, CUS procedures were maintained. Depression-like behaviors were assessed using the sucrose preference test (SPT), tail suspension test (TST), and open field test (OFT). Dynamic changes in neuroinflammation were monitored via longitudinal [18F]DPA-714 positron emission tomography (PET) scans at baseline and after 2 and 4 weeks of treatment. Ex vivo analyses included immunofluorescence quantification of hippocampal microglia (ionized calcium-binding adaptor molecule 1, Iba1), astrocytes (glial fibrillary acidic protein, GFAP), and 18 kDa translocator protein (TSPO) co-expression, alongside quantitative polymerase chain reaction (qPCR) and Western blotting for inflammatory markers (IL-1β, IL-4, TSPO). **Results**: MaR1 treatment selectively alleviated depression-like behaviors, significantly reversing CUS-induced anhedonia in the SPT and improving locomotor activity, while its effect on despair-like behavior (TST) was not statistically significant. Longitudinal PET imaging revealed a biphasic neuroimmune response, characterized by an initial increase in [18F]DPA-714 standardized uptake value (SUV) at 2 weeks, followed by a return toward baseline at 4 weeks. Histologically, MaR1 reversed CUS-induced hippocampal microglial loss, resulting in a rebound of microglial numbers, and normalized astrocytic activation. At the molecular level, MaR1 dynamically modulated cytokine expression, culminating in a significant upregulation of the pro-resolving marker IL-4 and TSPO at 4 weeks. **Conclusions**: These findings indicate that Maresin-1 treatment is associated with behavioral improvement and dynamic modulation of glial activity and TSPO PET signals in the hippocampus. This study highlights the value of TSPO PET imaging for monitoring dynamic glial changes during therapeutic intervention and provides supportive evidence for targeting neuroimmune pathways in depression.

## 1. Introduction

Depression (Major Depressive Disorder, MDD) is a pervasive and debilitating psychiatric disorder characterized by persistent low mood, anhedonia, and cognitive impairments, affecting over 350 million individuals worldwide and imposing a substantial global health burden [1]. Current first-line pharmacological treatments, such as selective serotonin reuptake inhibitors (SSRIs), are hampered by delayed onset of action, variable efficacy, and adverse side effects. This situation underscores the urgent need to elucidate novel pathophysiological mechanisms and develop more effective therapeutic strategies [2]. Accumulating evidence positions neuroinflammation as a pivotal contributor to the etiology and progression of depression [3]. Anti-inflammatory interventions have demonstrated promising antidepressant effects in both clinical and preclinical settings, reinforcing the inflammatory hypothesis of depression [4]. Within this framework, specialized pro-resolving mediators (SPMs)—a class of endogenous lipid mediators biosynthesized from omega-3 polyunsaturated fatty acids (n-3 PUFAs)—have emerged as critical regulators that actively terminate inflammation and promote tissue repair without immunosuppression [5,6]. Beyond resolving self-limiting acute inflammation, SPMs are increasingly recognized for their potential in mitigating chronic low-grade inflammation, a hallmark of several neuropsychiatric disorders, including depression. 

Epidemiological and clinical studies have long associated higher n-3 PUFA intake with reduced risk and severity of depression [7]. Additionally, supplementation with n-3 PUFAs alleviates depressive symptoms in patients and stress-exposed animals [8,9]. Preclinical evidence further indicates that n-3 PUFAs protect against stress-induced neuronal atrophy in the prefrontal cortex [10] and attenuate lipopolysaccharide (LPS)-triggered hippocampal inflammation [11]. It has been proposed that SPMs may mediate the beneficial effects of n-3 PUFAs in mood regulation. For instance, altered peripheral blood levels of resolvin D1 have been reported in depressed individuals, suggesting a disruption in inflammation resolution pathways [12]. In rodent models, several SPMs—including resolvins D1, D2, and E series—rapidly ameliorate LPS-induced depression-like behaviors [13,14]. Similarly, MaR1, a macrophage-derived mediator biosynthesized from docosahexaenoic acid (DHA), exerts robust anti-inflammatory, neuroprotective, and tissue-reparative effects in models of cerebral ischemia [15], sepsis [16], and pulmonary fibrosis [17]. In the central nervous system (CNS), MaR1 has been shown to suppress microglial activation, promote a pro-resolving microglial phenotype, restore synaptic plasticity, and improve cognitive function in Alzheimer’s disease models [18]. Given that microglial dysregulation and impaired inflammation resolution are implicated in depression pathophysiology, we hypothesized that MaR1 may hold therapeutic potential for depression through its dual actions in resolving neuroinflammation and promoting neural resilience. While our recent work demonstrated that MaR1 alleviated LPS-induced acute depression-like behavior [19], its effects in a chronic stress model, which better recapitulates the protracted nature of human depression and the associated in vivo neuroimmune dynamics, remain largely unexplored.

Microglia, the resident immune cells of the CNS, are central regulators of neuroinflammation and brain homeostasis, participating in synaptic pruning, cytokine release, and metabolic support [20]. Their functional states exist along a dynamic continuum, and both excessive activation and dystrophic depletion have been linked to depression pathogenesis [21,22]. Thus, longitudinal monitoring of microglial activity in vivo could provide critical insights into their role in depression progression and treatment response. The 18-kDa translocator protein (TSPO), primarily expressed on the outer mitochondrial membrane of activated microglia and astrocytes in the CNS, serves as a sensitive molecular imaging biomarker for glial activation and neuroinflammatory status [23]. Positron emission tomography (PET) with TSPO-targeted radiotracers enables non-invasive, longitudinal assessment of neuroinflammation in living subjects, offering unique insights into disease mechanisms and treatment effects [24,25]. Among second-generation TSPO tracers, [18F]DPA-714 exhibits high binding affinity, favorable pharmacokinetics, and a longer half-life suitable for extended imaging protocols [26,27]. Our group has previously validated the utility of [18F]DPA-714 PET in tracking hippocampal glial activation in a chronic unpredictable stress (CUS) model of depression [28].

Based on these considerations, the present study aimed to determine whether MaR1 treatment alleviates depression-like behaviors in a chronic unpredictable stress (CUS) mouse model and whether these behavioral changes correlate with dynamic alterations in hippocampal neuroimmune activity, as assessed via longitudinal [18F]DPA-714 PET imaging. By combining behavioral tests, in vivo PET, immunohistochemistry, and molecular analyses, we sought to delineate the temporal dynamics of MaR1’s effects on neuroinflammation and behavior, thereby advancing the understanding of pro-resolving lipid mediators as potential therapeutics for depression.

## 2. Materials and Methods

### 2.1. Animals

Four- to six-week-old male C57BL/6J mice (10–15 g) were obtained from the Laboratory Animal Center of Chongqing Medical University (Chongqing, China). Animals were acclimatized for one week under standard housing conditions (12-h light/dark cycle, lights on 08:00–20:00; temperature 22 ± 2 °C; humidity 55 ± 10%; group-housed, 5 mice per cage) with ad libitum access to food and water. After acclimatization, mice with baseline sucrose preference < 65% were excluded (n = 13). The remaining animals were randomly assigned to either the chronic unpredictable stress (CUS) group (n = 127) or the non-stressed control group (n = 40). The CUS group was singly housed and subjected to a 5-week stress protocol, whereas control mice remained group-housed under standard conditions without stress. After 5 weeks, mice in the CUS group were screened using the sucrose preference test (SPT). Animals showing a statistically significant reduction in sucrose preference compared to the contemporaneous control group (*p* < 0.05), with a decrease ≥ 20% relative to the control mean, were classified as “stress-susceptible” and included in subsequent pharmacological intervention. Mice not meeting these criteria were classified as “stress-resilient” and were not used further; they were euthanized in accordance with institutional animal welfare guidelines.

Fifty stress-susceptible mice were randomly allocated to two treatment groups: CUS/MaR1 group (treatment with Maresin1, 5 ug/kg, n = 25) and CUS/PBS group (treatment with PBS, n = 25). Treatment lasted 4 weeks, during which CUS procedures were maintained to sustain the depressive-like phenotype. All experimental protocols were reviewed and authorized by the Animal Research Ethics Committee of the First Affiliated Hospital of Chongqing Medical University, and were conducted in strict accordance with the guidelines outlined in the NIH Guide for the Care and Use of Laboratory Animals.

### 2.2. Chronic Unpredictable Stress (CUS) Protocol

The CUS protocol was conducted following established methods [28]. Mice were subjected to a variable regimen of 2–4 stressors per day, presented in an unpredictable order. These stressors included: food and water deprivation for 12 h, wet bedding for 24 h, cage tilting at a 45° angle for 12 h, disruption of the light cycle with 3 h of illumination during the dark phase, cage shaking for 12 h, intermittent strobe light exposure for 12 h, foot shock at 0.8 mA for 1 min, restraint stress for 3 h, and cold stress at 4 °C for 20 min. Control animals were maintained undisturbed in their standard home cages. Throughout the 5-week stress induction period, weekly measurements of body weight and sucrose preference were recorded.

### 2.3. Behavioral Tests

All behavioral tests were conducted between 09:00 and 14:00 by experimenters blinded to group assignment.

#### 2.3.1. Sucrose Preference Test (SPT)

The SPT was employed to assess anhedonia, a core depressive symptom. The test was administered weekly at 9:00 AM. Mice were individually housed in separate cages and provided with two identical, pre-weighed bottles—one containing sterile water and the other containing a 1% sucrose solution. Adequate food was supplied to minimize disturbance over a 24-h period. The placement of the sucrose bottle (left or right) was randomized. Sucrose preference was quantified as the percentage of sucrose solution consumed relative to total fluid intake.

#### 2.3.2. Tail Suspension Test (TST)

The TST was conducted following established procedures. Each mouse was suspended 50 cm above the ground by securing adhesive tape approximately 1 cm from the tail tip for a duration of 6 min. Immobility time was recorded during the final 4 min by two observers blinded to group assignment. Immobility was defined as the absence of any active movement while the animal hung passively. Mice that climbed their tails were excluded from subsequent analysis.

#### 2.3.3. Open Field Test (OFT)

The OFT was performed using a white square arena measuring 50 cm (length) × 50 cm (width) × 40 cm (height), with the floor subdivided into 25 equal 10 cm × 10 cm squares. Each mouse was gently placed in the center of the arena and allowed to explore freely for 5 min under dim, quiet conditions. Movement was recorded via an overhead camera. The arena was cleaned with 75% ethanol between trials to eliminate olfactory cues. Behavioral parameters, including total distance traveled, distance and time spent in the central versus peripheral zones, were analyzed using the SMART 3.0 Mouse Behavior Analysis System.

### 2.4. Maresin-1 Treatment Formula

Maresin-1 (Cayman Chemical, Ann Arbor, MI, USA) was dissolved in absolute ethanol (50 µg/500 µL), aliquoted under nitrogen, and stored at −80 °C. The sterile PBS solution was diluted to the concentration required for this experiment (1 µg/mL). Mice in the CUS/MaR1 group received 5 µg/kg MaR1 (i.p.) every other day for 4 weeks. The dose and duration of MaR1 administration were selected based on prior studies demonstrating biological efficacy in neuroinflammatory and behavioral models, as well as preliminary observations of tolerability and behavioral effects [19,28]. The treatment schedule was designed to encompass both early and delayed neuroimmune responses and to correspond with the longitudinal PET imaging framework. The CUS/PBS group received an equivalent volume of PBS. All injections were administered between 10:00 and 11:00.

### 2.5. Double Immunofluorescence

Ionized calcium-binding adaptor molecule-1 (Iba-1) and glial fibrillary acidic protein (GFAP) are used to label microglia and reactive astrocytes, respectively. Mice were euthanized by cervical dislocation immediately after the behavioral tests. Immunofluorescence staining was conducted following established protocols [29]. In brief, mice were anesthetized deeply with pentobarbital sodium and subjected to transcardial perfusion using 4% paraformaldehyde in 0.01 M phosphate buffer. Brain tissues were post-fixed in the same fixative and initially stored at 4 °C. Subsequently, the tissues underwent dehydration, paraffin embedding, and sectioning into slices of 3–5 μm thickness. After deparaffinization, sections were subjected to antigen retrieval by microwaving for 25 min in EDTA buffer (pH 8.0). Following this, sections were treated with 3% hydrogen peroxide and blocked with goat serum diluted in 3% bovine serum albumin (BSA; Servicebio, Wuhan, China) for 30 min. Tissue sections were then incubated overnight at 4 °C with primary antibodies: anti-TSPO (1:2000, Abcam, Cambridge, UK) combined with either anti-Iba-1 (1:1000, Novus Biologicals, Centennial, CO, USA) or anti-GFAP (1:2000, Abcam, Cambridge, UK). After washing with PBS, sections were exposed to secondary antibodies: fluorescein isothiocyanate (FITC)-conjugated goat anti-rabbit IgG (1:100) and tetramethylrhodamine isothiocyanate (TRITC)-conjugated goat anti-mouse IgG (1:100) (both from Servicebio, Wuhan, China) for 90 min at 37 °C. Following another PBS wash, nuclei were counterstained with DAPI for 10 min, and sections were mounted with coverslips. Fluorescence imaging was performed using a laser scanning confocal microscope (Leica Microsystems Heidelberg GmbH, Heidelberg, Germany) coupled with an Olympus SP2 inverted microscope equipped with a Fluoview FVX confocal scan head. Quantitative analysis was performed to determine the counts of Iba-1+, TSPO+, TSPO+/Iba-1+, GFAP+, and TSPO+/GFAP+ cells within the hippocampal region.

### 2.6. [18F]DPA-714 PET

Longitudinal PET/CT imaging was conducted using a Mediso Ltd. (Budapest, Hungary) system at three time points: baseline (prior to treatment), 2 weeks, and 4 weeks following the start of treatment. Approximately 8.0 MBq of [18F]DPA-714 in 200 µL of PBS was administered to the mice via tail vein injection. After injection, the animals were returned to their home cages for a 40-min uptake period. Subsequently, anesthesia was induced with 2% isoflurane, and imaging was performed on a nanoScan PET/CT system (Mediso Ltd., Budapest, Hungary). Each imaging session consisted of a 20-min static PET acquisition followed by a low-dose CT scan (parameters: 500 µA, 80 kV, 98 µm resolution, 360° rotation in 220 steps) for anatomical co-registration. Throughout the procedure, respiratory rate and body temperature were continuously monitored using a dedicated small animal physiological monitoring system (M2M Imaging Corp., Cleveland, OH, USA). All list-mode PET data were reconstructed using a 2D OSEM algorithm (frame structure: 4 × 30, 5 × 150, 6 × 450 s) without applying attenuation correction. The reconstructed PET images were then co-registered to a standard murine MRI template using PMOD software (version 3.7, PMOD Technologies, Zurich, Switzerland; www.pmod.com (accessed on 20 February 2022)). Volumes of interest (VOIs) were defined for the cortex, hippocampus, and amygdala. The uptake of [18F]DPA-714 was presented as a standardized uptake value (*SUV*), which was used to evaluate within-subject relative longitudinal changes, and was calculated from the 0–20 min post-scan onset interval (starting 40 min post-injection), using the following formula:

$$\mathrm{SUV}=\frac{A\;(\mathrm{kBq}/{\mathrm{cm}}^{3})}{\mathrm{ID}\;(\mathrm{kBq})/\mathrm{BW}\;\left(g\right)}$$

where *A* represents the tissue activity concentration, *ID* denotes the injected dose, and *BW* indicates the body weight.

### 2.7. Quantitative Real-Time PCR

Mice were humanely euthanized via cervical dislocation, and fresh brain tissue specimens were promptly harvested. These tissue samples were instantly flash-frozen in liquid nitrogen and subsequently maintained at −80 °C for subsequent real-time PCR analysis. Total RNA was isolated from hippocampal tissue employing the total RNA tissue extraction kit (Tiangen Biotech, Beijing, China), adhering strictly to the manufacturer’s protocol. RNA concentration was quantified using a NanoDrop 2000 spectrophotometer (Thermo Fisher Scientific, Waltham, MA, USA). Complementary DNA (cDNA) synthesis was carried out through reverse transcription with a dedicated kit (Servicebio, Wuhan, China). Quantitative real-time PCR was conducted on an ABI Prism 7500 real-time PCR system(Applied Biosystems, Foster City, CA, USA). Gene expression levels were normalized to the housekeeping gene *Gapdh* and relative quantification was calculated using the 2^−ΔΔCt^ method. The specific primer sequences utilized are detailed in Table 1.

### 2.8. Western Blot

Western blotting was conducted following established protocols. Brain tissue homogenates were prepared on ice using RIPA lysis buffer supplemented with PMSF (Beyotime, Haimen, China) to inhibit proteolysis. Following centrifugation at 12,000 rpm for 10 min at 4 °C, the supernatant was collected. Protein concentration was quantified using the Enhanced BCA Protein Assay Kit (Beyotime, Haimen, China). Subsequently, 5× loading buffer was mixed with the supernatant, and the samples were heated in boiling water for 5 min before storage at −80 °C. For electrophoresis, 50 μg of total protein per sample was loaded onto a polyacrylamide gel and separated by SDS-PAGE using a 5% stacking gel at 75 V and a 10% resolving gel at 120 V. Proteins were then transferred onto a polyvinylidene fluoride (PVDF) membrane with a pore size of 0.45 μm (Millipore, Billerica, MA, USA) at a constant current of 300 mA for 60 min. Uniform protein loading and transfer efficiency were verified by Ponceau S staining. The membrane was blocked with 5% skim milk at room temperature for 30 min to minimize nonspecific binding, followed by overnight incubation at 4 °C with primary antibodies targeting TSPO (1:1000), IL-1β (1:1000), and ACTIN (1:1000). After three 5-min washes with Tween-20-Tris-buffered saline (TTBS), the membrane was incubated with a horseradish peroxidase-conjugated secondary antibody (goat anti-rabbit IgG-HRP, 1:9000; Zhongshan Golden Bridge, Zhongshan, China) for 30 min at room temperature and washed again under the same conditions. Protein bands were detected using an enhanced chemiluminescence substrate kit (Beyotime Institute of Biotechnology, Shanghai, China) and imaged with a digital scanner (Bio-Rad Laboratories). Band intensity was quantified by measuring optical density (OD) values using grayscale analysis software (Alpha Innotech, version 4.0.0, San Leandro, CA, USA).

### 2.9. Statistical Analysis

Data are presented as mean ± SD. Statistical analyses were performed using GraphPad Prism 8.4.3 (GraphPad Software, San Diego, CA, USA). Normality was assessed using the Shapiro–Wilk test, and homogeneity of variance was evaluated using the Brown–Forsythe test. For single-time-point comparisons among three groups, one-way ANOVA followed by Tukey’s post hoc test was used when assumptions were met. If variances were unequal, Welch’s ANOVA with Dunnett’s T3 post hoc test was applied. Two-way repeated-measures ANOVA (time × group) followed by Šidák’s multiple comparisons test. For longitudinal PET SUV data, acquired from the same animals at multiple time points, one-way repeated-measures ANOVA followed by Tukey’s test was applied only for within-group comparisons across time points. All tests were two-tailed, and *p* < 0.05 was considered statistically significant. Experimenters were blinded to group allocation throughout data collection and analysis.

## 3. Results

### 3.1. MaR1 Treatment Alleviates Depression-like Behavior with Differential Efficacy Across Paradigms

**Figure 1:**
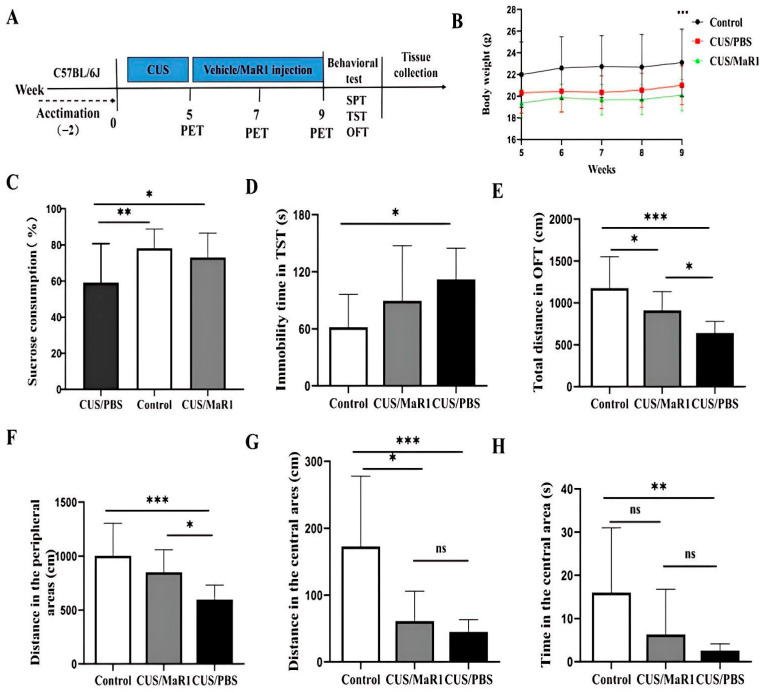
Experimental procedures and results of behavioral experiments. (**A**) Experimental procedures; (**B**) Body weight changes; (**C**) Sucrose preference percentage (%); (**D**) Immobility time in the TST; (**E**) Total distance traveled in OFT; (**F**) Distance traveled in the peripheral area in the OFT; (**G**) Distance traveled in the central area in the OFT; (**H**) Time spent in the central area in the OFT. All data are expressed as the mean ± SD. Body weight across weeks (**B**) was analyzed using two-way repeated-measures ANOVA (time × group), followed by Sidak’s multiple-comparisons test when appropriate. Panels (**C**–**H**) were analyzed using one-way ANOVA followed by Tukey’s multiple-comparisons test (or Welch’s ANOVA followed by Dunnett’s T3 test when variance homogeneity was not met). * *p* < 0.05, ** *p* < 0.01, *** *p* < 0.001; “ns” indicates not significant. The brackets denote the specific pairwise comparisons.

Mice subjected to the chronic unpredictable stress (CUS) protocol showed significantly reduced body weight gain over the experimental period compared to non-stressed controls (*p* < 0.001, Figure 1B). MaR1 treatment (5 µg/kg, every other day for 4 weeks) did not alleviate the CUS-induced weight loss, with no significant difference observed between the CUS/MaR1 and CUS/PBS groups (Figure 1B). In behavioral tests assessing distinct depressive-like domains, MaR1 demonstrated selective efficacy. In the sucrose preference test (SPT), a measure of anhedonia, MaR1-treated mice displayed a significantly higher sucrose preference compared to the PBS-treated stressed controls (*p* = 0.032, Figure 1C). Their preference reached levels comparable to the non-stressed control group (*p* = 0.75, Figure 1C). In contrast, sucrose preference in the CUS/PBS group remained significantly lower than in controls (*p* = 0.007, Figure 1C). The effects of MaR1 on despair-like behavior, as measured by the tail suspension test (TST), were more limited. Although a numerical reduction in immobility time was observed in the CUS/MaR1 group relative to the CUS/PBS group, this difference did not reach statistical significance (*p* = 0.231, Figure 1D). As expected, CUS/PBS mice exhibited significantly greater immobility than controls (*p* = 0.01, Figure 1D). Assessment of locomotor activity and exploratory behavior in the open field test (OFT) revealed partial improvements. MaR1-treated mice traveled a significantly greater total distance (*p* = 0.04, Figure 1E) and distance in the peripheral zone (*p* = 0.024, Figure 1F) compared to the CUS/PBS group. However, both the CUS/MaR1 and CUS/PBS groups exhibited reduced activity in both total and central zones compared to non-stressed controls, indicating that MaR1 only partially normalized CUS-induced locomotor and exploratory deficits.

### 3.2. Longitudinal [18F]DPA-714 PET Imaging Reveals a Dynamic Neuroimmune Response to MaR1

**Figure 2:**
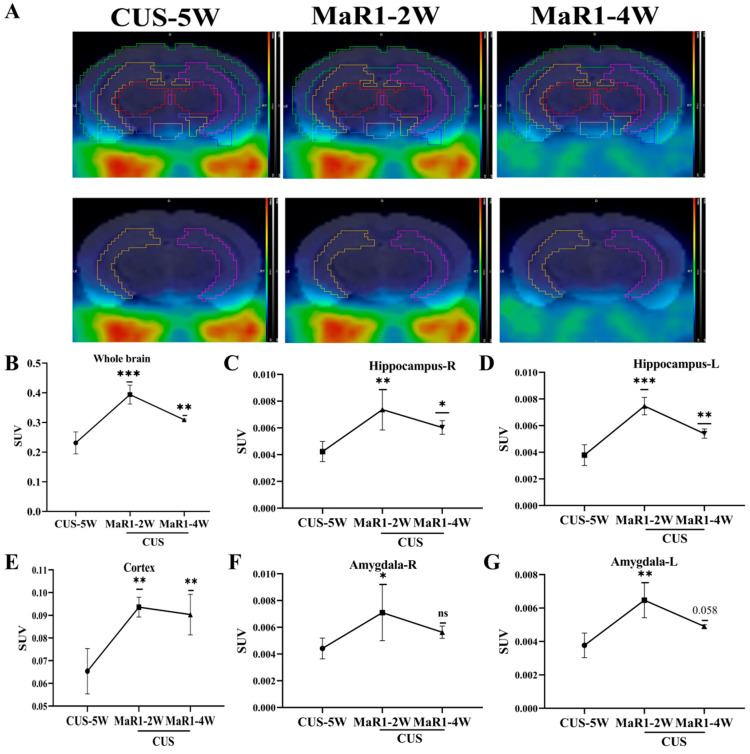
MaR1 treatment induces dynamic changes of [18F]DPA-714 signals in vivo. (**A**) Representative PET/CT mouse brain images (sagittal, coronal, and transverse) at CUS-5W, MaR1-2W, and MaR1-4W; (**B**–**G**) Quantitative analysis of [18F]DPA-714 in regions of interest, such as whole brain, hippocampus, amygdala, and cortex. The results represent normalized MRI images derived from individual images. All data are expressed as the mean ± SD. Longitudinal PET SUVs (CUS-5W vs. MaR1-2W vs. MaR1-4W) were analyzed using one-way repeated-measures ANOVA, followed by Tukey’s multiple-comparisons test. * *p* < 0.05, ** *p* < 0.01, *** *p* < 0.001; exact *p* values are shown when indicated in the panels (e.g., *p* = 0.058). Brackets denote the specific pairwise comparisons between time points. The color scale represents the standardized uptake value (*SUV*), with warmer colors (yellow–red) indicating higher tracer uptake and cooler colors (blue–green) indicating lower uptake.

To dynamically monitor in vivo neuroinflammatory changes, a subset of mice from the CUS/MaR1 group (n = 5) underwent longitudinal [18F]DPA-714 PET scans at baseline (pre-treatment) and after 2 and 4 weeks of MaR1 intervention. Representative PET/MRI fusion images are shown in Figure 2A. Analysis of standardized uptake values (SUVs) revealed a time-dependent and biphasic response. After 2 weeks of MaR1 treatment, a significant increase in [18F]DPA-714 SUV was observed in multiple brain regions, including the hippocampus, amygdala, and prefrontal cortex (PFC) (Figure 2B–G). Notably, this elevation was transient, as by 4 weeks of treatment, SUVs in these regions had declined, returning to levels approaching the pre-treatment baseline (Figure 2B–G). This outcome signifies the successful reversal of the CUS intervention-induced decline in [18F]DPA-714 SUV within the brain.

### 3.3. MaR1 Reverses CUS-Induced Reductions in Hippocampal Microglia and Modulates TSPO Expression

**Figure 3:**
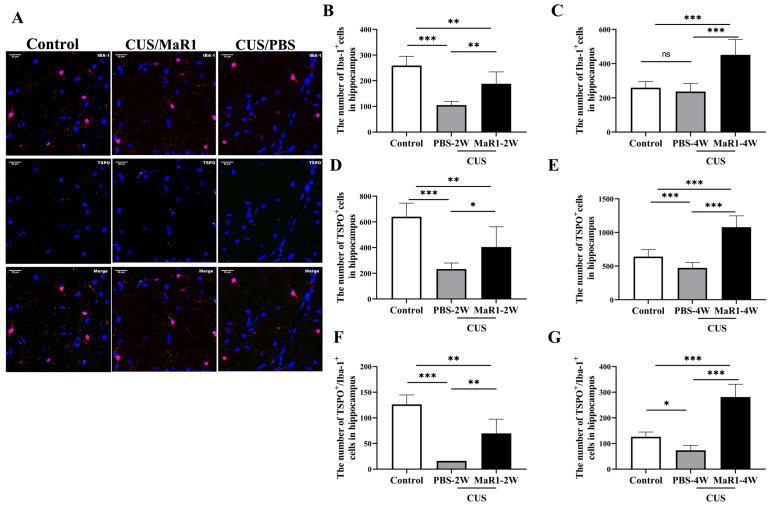
Effects of MaR1 treatment on the number of microglia and TSPO+ cells in the hippocampus. (**A**) Representative images of immunofluorescence staining with anti-TSPO and anti-Iba-1 antibodies; blue indicates nuclei stained with DAPI, red indicates Iba-1–positive microglia, and green indicates TSPO immunoreactivity. Merged images illustrate the co-localization of Iba-1 and TSPO. (**B**,**C**) Quantification of Iba-1+ cells in the hippocampus; (**D**,**E**) Quantification of TSPO+ cells in the hippocampus; (**F**,**G**) Quantification of TSPO+/Iba-1+ cells in the hippocampus. All data are expressed as the mean ± SD. For each time point (2W or 4W), group differences were analyzed using one-way ANOVA followed by Tukey’s multiple-comparisons test. * *p* < 0.05, ** *p* < 0.01, *** *p* < 0.001; “ns” indicates not significant. Brackets denote the specific pairwise comparisons.

Given the pronounced SUV changes in the hippocampus, we performed histological analyses in this region. Immunofluorescence staining for Iba1 (microglia marker) and TSPO was quantified (Figure 3A). Consistent with our previous findings, 5 weeks of CUS exposure significantly reduced the number of Iba1^+^ microglia in the hippocampus compared to controls. After 2 weeks of treatment, the CUS/MaR1-2W group showed a significant increase in the number of Iba-1+ cells (*p* = 0.003), TSPO+ cells (*p* = 0.034), and TSPO+/Iba-1+ cells (*p* = 0.001) compared to the CUS/PBS-2W group (Figure 3B,D,F). However, at this time point, both stressed groups still had fewer Iba-1+ and TSPO+ cells than controls. Notably, after 4 weeks of MaR1 treatment, this trend was amplified. The CUS/MaR1-4W group exhibited significantly higher numbers of Iba-1+ cells, TSPO+ cells, and TSPO+/Iba-1+ cells than both the control group (*p* < 0.001) and the CUS/PBS-4W group (*p* < 0.001) (Figure 3C,E,G). In contrast, the CUS/PBS-4W group remained significantly below control levels (Figure 3E,G). These findings indicate that prolonged MaR1 treatment not only prevented CUS-induced microglial loss but also promoted a significant rebound in microglial number and TSPO co-expression beyond physiological levels.

### 3.4. MaR1 Normalizes CUS-Induced Astrocytic Activation in the Hippocampus

**Figure 4:**
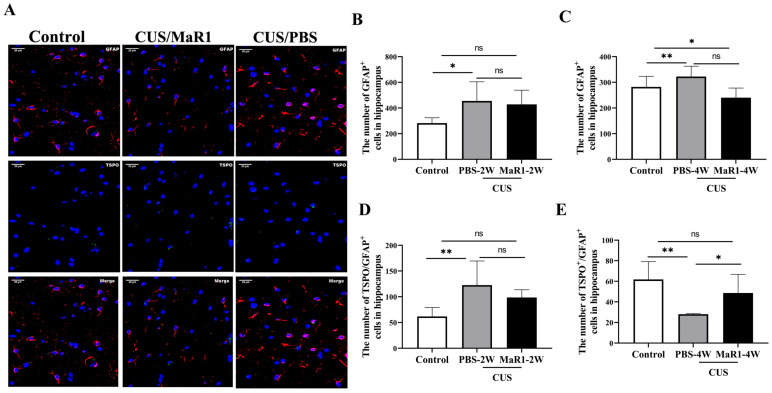
Effects of MaR1 treatment on GFAP+ astrocytes in the hippocampus. (**A**) Representative images of immunofluorescence staining with anti-GFAP and anti-TSPO antibodies; (**B**,**C**) Quantification of GFAP+ cells in the hippocampus; (**D**,**E**) Quantification of TSPO+ /GFAP+ cells in the hippocampus; All data are expressed as the mean ± SD. For each time point, group differences were analyzed using one-way ANOVA followed by Tukey’s multiple-comparisons test. * *p* < 0.05, ** *p* < 0.01; “ns” indicates not significant. Brackets denote the specific pairwise comparisons.

We next assessed the effects on astrocytes using GFAP and TSPO immunofluorescence (Figure 4A). After 2 weeks, the number of GFAP+ astrocytes remained elevated in both stressed groups compared to controls, though only the CUS/PBS-2W group reached statistical significance (Figure 4B). A striking reversal was observed at the 4-week time point. The number of GFAP+ astrocytes in the CUS/MaR1-4W group was significantly lower than in the control group (*p* = 0.029, Figure 4C) and showed a strong decreasing trend compared to the CUS/PBS-4W group (*p* = 0.051, Figure 4C). Conversely, astrocyte numbers in the CUS/PBS-4W group remained markedly higher than in controls (*p* = 0.001, Figure 4C). Analysis of TSPO^+^/GFAP^+^ cells further supported this pattern, showing a significant increase in the CUS/MaR1-4W group compared to the CUS/PBS-4W group (*p* = 0.045, Figure 4E). Together, these data suggest that while CUS sustained astrocytic activation (GFAP+), MaR1 treatment over 4 weeks promoted a reduction in overall astrocyte numbers and an increase in the TSPO-expressing astrocyte subset.

### 3.5. Dynamic Effects of MaR1 on Hippocampal Inflammatory and Neuroimmune-Related Markers Following Chronic Unpredictable Stress

**Figure 5:**
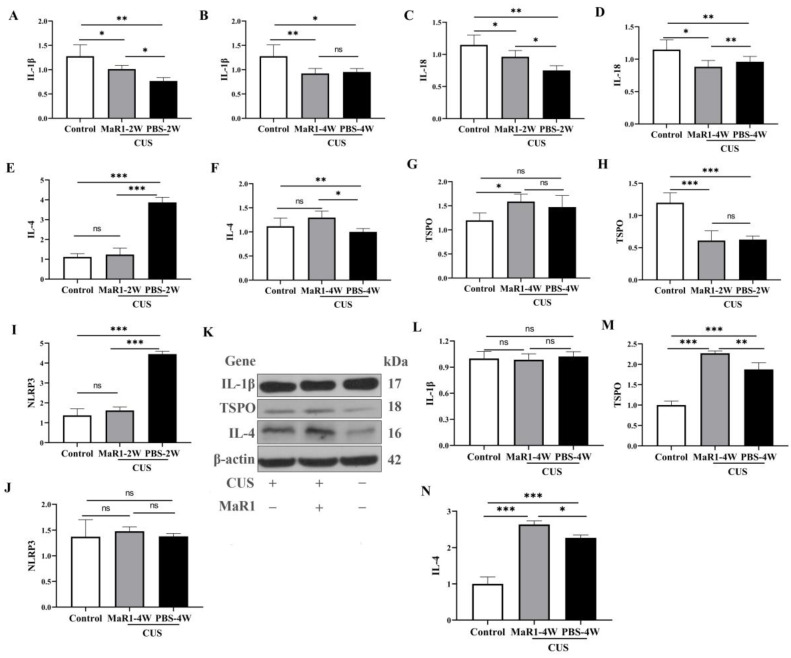
Effects of MaR1 treatment on pro-inflammatory and anti-inflammatory cytokines. (**A**,**B**) RNA Expression of *Il1b* in the hippocampus; (**C**,**D**) RNA Expression of *Il18* (RT-PCR) in the hippocampus; (**E**,**F**) RNA Expression of *Il4* in the hippocampus; (**G**,**H**) RNA Expression of *Tspo* in the hippocampus; (**I**,**J**) RNA Expression of *Nlrp3*. (**K**) Western blot analysis of protein expression levels of IL-1β, TSPO, and IL-4 in the hippocampus; (**L**–**N**) Quantitative analysis of hippocampal IL-1β (**L**), TSPO (**M**), and IL-4 (**N**) protein levels after 4 weeks of treatment. All data are expressed as the mean ± SD. Within each panel, comparisons among the three groups were analyzed using one-way ANOVA followed by Tukey’s multiple-comparisons test. * *p* < 0.05, ** *p* < 0.01, *** *p* < 0.001; “ns” indicates not significant. Brackets denote the specific pairwise comparisons.

To systematically characterize the temporal dynamics of the neuroimmune state under MaR1 treatment, we performed time-course analyses of both mRNA and protein expression of key cytokines and TSPO in hippocampal tissue. Pro-inflammatory cytokines showed a complex temporal pattern. At the mRNA level, *Il1b* expression was significantly higher in the CUS/MaR1-2W group than in the CUS/PBS-2W group (*p* = 0.043, Figure 5A). However, both stressed groups at 2 and 4 weeks maintained lower *Il1b* and *Il18* mRNA levels compared to the non-stressed control group (Figure 5A–D). Western blot analysis of protein levels mirrored these trends. No significant differences were observed in IL-1β protein levels among the CUS/MaR1-4W group, the CUS/PBS-4W group, and the control group (Figure 5L).

In contrast, markers associated with inflammation resolution were upregulated by MaR1. *Il4* mRNA expression, which was suppressed in the CUS/PBS-2W group, showed a significant increase in the CUS/MaR1-4W group compared to the CUS/PBS-4W group (*p* = 0.011, Figure 5F), with a strong upward trend versus controls (*p* = 0.085, Figure 5F). This increase was confirmed at the protein level by Western blot in the CUS/MaR1-4W group compared to the CUS/PBS-4W group (*p* = 0.037, Figure 5N).

TSPO expression was dynamically regulated. *Tspo* mRNA was low in both stressed groups at 2 weeks but became significantly higher in the CUS/MaR1-4W group than in both the control (*p* = 0.017, Figure 5G) and CUS/PBS-4W groups (Figure 5G). Consistent with mRNA and immunofluorescence data, Western blot analysis confirmed a significant increase in TSPO expression in the CUS/MaR1-4W group compared to the CUS/PBS-4W group (*p* = 0.013, Figure 5M).

NLRP3 inflammasome component expression was altered by stress but not further modulated by MaR1 at the endpoint. *Nlrp3* mRNA was elevated in the CUS/PBS-2W group but returned to control levels in all groups by 4 weeks, with no significant effect of MaR1 (Figure 5I).

The RNA expression of *Tspo* in the hippocampus was significantly lower in both the CUS/MaR1-2W group and the CUS/PBS-2W group when compared to the Control group (*p* < 0.001, Figure 5H). However, in the CUS/MaR1-4W group, *Tspo* expression was higher compared to the Control group (*p* = 0.017, Figure 5G). These findings indicate that MaR1 treatment significantly increased the level of *Tspo* in the hippocampus, effectively reversing the decrease in *Tspo* expression caused by CUS intervention. The RNA expression of *Nlrp3* in the hippocampus was significantly lower in both the CUS/MaR1-2W group and the Control group when compared to the CUS/PBS-2W group (*p* < 0.001, Figure 5I). However, there was no significant difference among the CUS/MaR1-4W group, CUS/PBS-4W group, and Control group.

## 4. Discussion

In this study, we combined longitudinal [18F]DPA-714 PET imaging with multimodal analyses to investigate the neuroimmune and behavioral effects of Maresin-1 (MaR1) in a mouse model of CUS-induced depression. In behavioral tests, MaR1 administration resulted in a significant increase in sucrose preference and enhanced locomotor activity in the open field test, while a non-significant trend toward decreased immobility was noted in the tail suspension test. These behavioral findings indicate that the effects of MaR1 appear more pronounced in measures of anhedonia and motivation compared to those assessing despair-like behavior. The principal findings indicate that MaR1 treatment alleviates specific depression-like behaviors, while eliciting a dynamic, biphasic neuroimmune response. This response is characterized by an early elevation and subsequent normalization of TSPO PET signal, coupled with a sustained restoration of hippocampal microglial density. Collectively, these findings position MaR1 not as a broad-spectrum anti-inflammatory agent in this chronic stress context, but as a modulator of glial homeostasis that promotes the recovery of stress-sensitive behaviors and the depleted microglial population.

Microglial loss in the hippocampus is recognized as a critical pathophysiological change contributing to the development of depression. In our study, we observed that the number of microglia, TSPO^+^ cells, and TSPO-expressing microglia in the hippocampus exhibited a significant “time-dependent” increase after Maresin1 treatment, indicating a reversal of stress-induced microglial depletion and a restoration of microglial homeostasis. This effect reversed the microglial loss in the hippocampus induced by CUS intervention. Consequently, we hypothesize that Maresin1 alleviates depression at least in part by promoting microglial homeostasis through reversing microglial loss induced by CUS intervention. Our finding aligns with previous research. For instance, studies by Kreisel et al. [21], Tong et al. [22], and Gong et al. [30] have all reported that agents such as LPS and M-CSF can alleviate depression-like behavior in rodents by reversing microglial loss in the hippocampus induced by CUS intervention. Additionally, these studies have suggested that either blocking excessive microglial activation or reversing microglial loss induced by chronic stress may represent potential strategies for ameliorating depression [30]. The antidepressant effect of LPS in CUS-exposed mice partly elucidates the antidepressant effect of LPS observed in severely depressed patients [31]. Recent studies have also demonstrated that a single injection of M-CSF can rapidly and persistently improve depression-like behavior induced by CUS in mice, potentially via activation and restoration of hippocampal microglia [32]. Furthermore, Gao et al. found that amphotericin B liposomes exerted an antidepressant effect by reversing CUS-induced reduction in microglia in the dentate gyrus (DG) [33]. Thus, the notion of categorizing microglial function into a simple dichotomy of “pro-inflammatory” or “anti-inflammatory” to distinguish between “good” and “bad” microglia is increasingly being challenged. Instead, the microglial response to stress and injury appears to be a complex and dynamic process that cannot be simplified into such rigid categories [34]. This highlights the importance of microglial homeostasis in the onset and progression of depression. In summary, our study reinforces the idea that microglia play a pivotal role in depression and suggests that maintaining microglial homeostasis may be crucial for managing the condition.

Our study also uncovered an important dissociation between cellular changes and PET imaging findings. Specifically, although the number of microglia and TSPO-expressing microglia in the hippocampus displayed a “time-dependent” increase following Maresin1 treatment, the hippocampal [18F]DPA-714 SUV did not follow a parallel trajectory and instead declined after 4 weeks of treatment compared with the 2-week time point. This temporal mismatch suggests that longitudinal TSPO PET signals cannot be interpreted as a direct surrogate for microglial abundance alone. To further explore potential contributors to this dissociation, we examined astrocytic changes in the hippocampus and observed a significant decrease in astrocyte number after 4 weeks of Maresin1 treatment. This reduction may partially contribute to the observed decline in TSPO PET signal at the later time point. However, given that TSPO is expressed by multiple CNS and vascular-associated cell populations, and that we did not assess endothelial cells, infiltrating peripheral immune cells, or blood–brain barrier integrity, cell-type–specific attribution of SUV changes remains inherently limited. Accordingly, the dynamic changes in [18F]DPA-714 SUV during Maresin1 treatment are more appropriately interpreted as reflecting an integrated neuroimmune response involving multiple TSPO-expressing cell populations, rather than changes in a single glial subtype. This highlights the sensitivity of TSPO PET for longitudinal monitoring of neuroimmune dynamics in vivo, while also underscoring its limitations with respect to cellular specificity. We therefore explicitly acknowledge this methodological limitation and emphasize that future studies will need to incorporate complementary approaches such as CD45/CCR2 immunostaining, assessments of blood–brain barrier integrity, or cell-type–specific isolation strategies to further delineate the relative contributions of distinct cellular populations to dynamic TSPO PET signals. Moreover, these findings emphasize the complex interplay between microglia and astrocytes in neuroimmune remodeling and their potential roles in depression.

As discussed above, substantial evidence highlights Maresin-1 as a specialized pro-resolving mediator (SPM) with potent anti-inflammatory and inflammation-resolving properties [15]. These effects have been extensively characterized in cellular and animal models involving macrophage or microglial activation. In the present study, rather than directly investigating lipid metabolic pathways, our data provide in vivo evidence that Maresin-1 treatment is associated with the reversal of stress-induced microglial loss and the restoration of microglial homeostasis, as captured by longitudinal TSPO PET imaging and histological analyses. Inflammation is a self-limiting and protective process that can restore tissue homeostasis under the coordinated actions of SPMs. Multiple pathways have been proposed to contribute to inflammation resolution, including macrophage or microglial phenotypic modulation, regulation of reactive oxygen species, and the production of catabolic mediators such as proteins, lipids, and gaseous molecules [35]. Among these mechanisms, the concept of a “class switch” of lipid mediators—whereby pro-inflammatory eicosanoids such as prostaglandins and leukotrienes give rise to SPMs has been proposed based primarily on prior biochemical and immunological studies. It should be emphasized that the present study did not quantify upstream lipid mediators (e.g., PGE_2_, LTB_4_) or key metabolic enzymes (e.g., COX-2, 5-LOX) in the hippocampus; therefore, no direct experimental evidence is provided to support lipid mediator class switching in this model. Accordingly, any discussion of lipid mediator class switching in the context of Maresin-1 treatment should be regarded as a literature-based and speculative hypothesis rather than a demonstrated mechanism. SPMs exert their biological actions through receptor-dependent signaling pathways, predominantly involving G protein–coupled receptors. Microglia have been shown to express multiple SPM-related receptors, including ALX/FPR2 and GPR32/DRV1 [5]. In addition, Maresin-1 has been reported to activate surface LGR6 in astrocytes and neurons, as well as intracellular RORα, which is expressed in most brain cell types except oligodendrocytes [36]. Within this framework, our findings raise the possibility that restoration of microglial populations may re-establish cellular responsiveness to SPM signaling, thereby contributing to improved neuroimmune regulation and behavioral outcomes. However, this interpretation remains indirect and requires further experimental validation. In summary, the current results support a role for Maresin-1 in modulating neuroimmune status and behavioral phenotypes in CUS-exposed mice, primarily through the restoration of microglial homeostasis. Whether lipid mediator class switching contributes to these effects remains an open question and warrants direct investigation using lipidomics and pathway-specific approaches in future studies.

Microglia and astrocytes are pivotal regulators of neuroinflammation, and the NLRP3 inflammasome plays a central role in controlling the maturation and release of pro-inflammatory cytokines such as IL-1β and IL-18. In the present study, we observed a dynamic pattern of inflammatory signaling following Maresin-1 treatment. Administration of Maresin-1 for 2 weeks partially reversed the reduced hippocampal levels of IL-1β and IL-18 induced by CUS exposure, whereas prolonged treatment for 4 weeks was associated with a suppression of NLRP3 expression. Notably, the levels of these pro-inflammatory cytokines did not increase concomitantly with the recovery of microglial numbers, suggesting that restoration of microglial populations was not accompanied by exaggerated inflammatory activation. These findings indicate that Maresin-1 may exert anti-inflammatory effects while promoting microglial homeostasis under conditions of relatively low baseline neuroinflammation. This is consistent with previous reports describing its pro-resolving properties. In addition, we demonstrated that Maresin-1 treatment was associated with increased IL-4 and TSPO expression in the hippocampus at both the mRNA and protein levels, with Western blot analyses confirming the increased protein expression. Importantly, given that the comparison of IL-4 mRNA levels with controls did not reach statistical significance (*p* = 0.085), we interpret these findings as an association rather than evidence of a direct causal mechanism. IL-4 is widely regarded as an anti-inflammatory cytokine linked to M2-like microglial polarization and has been shown to modulate monoaminergic neurotransmission, including inhibition of the serotonin transporter. Robust evidence suggests that elevated levels of IL-4 and IL-10 are associated with antidepressant-like effects. Park et al. demonstrated that IL-4 attenuates depression-related behaviors by suppressing central glial activation, reducing neuroinflammation, and reversing IL-1β-induced neurotransmitter alterations [37]. Furthermore, IL-4 has been reported to induce Arg1 expression in astrocytes, thereby restoring hippocampal neurogenesis and enhancing resilience to stress-induced depression [38]. Maresin-1, a bioactive lipid mediator derived from DHA, exhibits potent anti-inflammatory and pro-resolving actions in a variety of pathological contexts, including traumatic brain injury, cerebral infarction, Alzheimer’s disease, and metabolic disorders. Previous studies have shown that Maresin-1 enhances macrophage phagocytic activity and promotes tissue remodeling by facilitating the clearance of apoptotic debris [39,40]. In models of Alzheimer’s disease, Maresin-1 has been reported to ameliorate cognitive impairment by reducing pro-inflammatory cytokines (TNF-α and IL-6) while increasing anti-inflammatory mediators such as IL-2 and IL-10 [18]. Moreover, Maresin-1 can promote polarization of CD11c^−^CD206^+^ (M2-like) macrophages [41], which may favor an anti-inflammatory milieu. Within this context, our data support the notion that Maresin-1–associated increases in IL-4 expression may contribute to improved neuroimmune balance and behavioral outcomes, although direct causal links remain to be established.

The translocator protein (TSPO) is primarily involved in cholesterol transport and steroidogenesis [42]. Neurosteroids are known to exert rapid anxiolytic and antidepressant effects [43]. Reduced TSPO expression and neurosteroid synthesis have been observed in multiple depression models, including learned helplessness and postpartum depression [44,45]. Overexpression of TSPO in the hippocampus produces antidepressant-like effects through increased tetrahydroprogesterone levels. Exogenous TSPO ligands improve depression-like behaviors via multiple mechanisms, including activation of mTOR signaling; restoration of synaptic plasticity; modulation of monoaminergic systems; and enhancement of 5-HT and BDNF signaling [46,47,48,49]. Consistent with these findings, our observation that Maresin-1 treatment is associated with increased hippocampal TSPO expression suggests a potential link between TSPO-related neurosteroid pathways and the behavioral improvements observed in this study, although this does not imply a direct mechanistic relationship.

In summary, the present results indicate that Maresin-1 modulates neuroimmune status and depression-related behaviors in CUS-exposed mice. This is associated with restoration of microglial homeostasis and increased IL-4 and TSPO expression. These findings do not establish MaR1 as a definitive antidepressant treatment but rather highlight its role in neuroimmune regulation relevant to stress-induced affective disturbances. Given the multifaceted actions of Maresin-1 at the intersection of inflammation and depression, further studies incorporating targeted genetic or pharmacological interventions are required to delineate the precise molecular pathways involved. Several important limitations must be acknowledged: (1) The use of male adolescent mice limits the translational relevance regarding females and other age groups. (2) Screening based primarily on sucrose preference may not capture the full heterogeneity of depression-like phenotypes, and the small sample size for PET imaging (n = 5/group) limits the statistical power of those analyses. (3) The sole reliance on standardized uptake value (*SUV*) is subject to considerable limitations. In contrast, reference tissue models, such as binding potential (BP) or distribution volume ratio (DVR), provide more specific indicators of translocator protein (TSPO) availability. Future investigations should therefore integrate these more refined kinetic modeling approaches. (4) The study design is correlative and descriptive, and the absence of interventions using TSPO antagonists, IL-4 knockout models, or microglial depletion techniques therefore precludes definitive causal conclusions regarding MaR1’s mechanism of action.

## 5. Conclusions

This study provides novel, integrative evidence that MaR1 exerts beneficial effects in a chronic stress model through dynamically modulating neuroimmune homeostasis, with a particular emphasis on reversing microglial loss. Its efficacy appears more pronounced in ameliorating motivational deficits than in ameliorating despair-like behavior. By adopting a dynamic, multi-modal approach, this study underscores the potential of pro-resolving lipid mediators as a promising therapeutic strategy designed to restore neural-immune communication in stress-related psychiatric disorders.

## Figures and Tables

**Table 1:** Primer sequences used for RT-PCR.

Name	Sequence (5′–3′)
*Tspo*	GCAGAAACCCTCTTGGCATC
	AGCGTCCTCTGTGAAACCTCC
*Il4*	TGTCATCCTGCTCTTCTTTCTCG
	TTTGGCACATCCATCTCCGT
*Il1b*	GCATCCAGCTTCAAATCTCGC
	TGTTCATCTCGGAGCCTGTAGTG
*Il18*	TGAAGTAAGAGGACTGGCTGTGA
	TTGGCAAGCAAGAAAGTGTCC
*Nlrp3*	TAAGAACTGTCATAGGGTCAAAACG
	GTCTGGAAGAACAGGCAACATG

## Data Availability

The original contributions presented in this study are included in the article. Further inquiries can be directed to the corresponding authors.

## Abbreviations

The following abbreviations are employed throughout this manuscript:

CUS	Chronic unpredictable stress
TSPO	The 18-kDa translocator protein
SUV	Standardized Uptake Value
IP	Intraperitoneal injection
SPT	Sucrose preference test
TST	Tail suspension test
OFT	Open field test
PET	Positron emission tomography
Iba	Ionized calcium-binding adaptor molecule
GFAP	Glial fibrillary acidic protein
